# Burnout in early year medical students: experiences, drivers and the perceived value of a reflection-based intervention

**DOI:** 10.1186/s12909-023-04948-0

**Published:** 2024-01-03

**Authors:** Mabel Prendergast, Alexandra M. Cardoso Pinto, Christopher-James Harvey, Elizabeth Muir

**Affiliations:** 1 School of Medicine, Imperial College London, London, UK; 2 School of Public Health, Imperial College London, London, UK; 3 Department of Primary Care and Public Health, Imperial College, London, UK

**Keywords:** Burnout, Cognitive fatigue, Emotional exhaustion, Reflection, Medical students

## Abstract

**Introduction:**

According to the 11th Revision of the International Classification of Diseases, burnout is defined as a syndrome resulting from chronic work-related stress that has not been successfully managed. Burnout is increasingly prevalent amongst medical students and has been shown to lead to worsened academic engagement, feelings of inadequacy, poor mental health and increased risk of withdrawal from the course. The aim of this study was to explore the experience of burnout amongst early year medical students and evaluate the perceived impact of a reflection-based intervention on their awareness and experience of burnout.

**Methods:**

The reflection-based intervention comprised two tutorials covering the presentation, drivers, impact and management strategies for burnout syndrome. These were introduced into the second-year medical curriculum at Imperial College London. As part of the reflection-based intervention, students were invited to complete an anonymous Qualtrics form three times during the academic year. This included the Shirom-Melamed Burnout Measure (SMBM) and a free-text question prompting the student to consider their stressors at the time of completing the intervention. The former is composed of 14-questions measuring the extent of feelings or behaviours suggestive of burnout, divided into three categories: physical fatigue, cognitive weariness and emotional exhaustion. At the end of the academic year, students were invited to participate in an online focus group to further explore their experience of burnout and their perceived value of the reflection-based intervention. Results of the SMBM were explored descriptively; free-text questions and the focus group transcript were analysed using inductive thematic analysis.

**Results:**

A total of 59 submissions for the reflection-based intervention were analysed: 26 students participated and consented in the first round, 8 in the second and 25 in the third round. Overall median burnout scores were 4 (IQR 3–5), 2 (IQR 1–4) and 3 (IQR 2–5) in each round of the SMBM, respectively. A total of 8 (30.8%) met the threshold for severe burnout (≥ 4.4) in round 1 of the questionnaire, zero in the second round and 4 (16%) in the third round. Physical and cognitive fatigue showed higher median scores than emotional exhaustion in every round. Four students participated in the focus group, which had two sections. The first was reflecting on burnout in medical school and the intervention, which revealed four themes: (1) indicators of burnout (often insidious, but may involve lack of energy and motivation, or changes in perceived personality); (2) perceived drivers of burnout (perceived expectation that medical school is supposed to be challenging and consistent prioritisation of work over wellbeing); (3) working habits of medical students (unachievable self-expectations and feelings of guilt when not working); (4) value of the intervention (the teaching and reflection-based intervention prompted students to identify signs of burnout in themselves and consider management strategies). The second section included considerations for implementing burnout interventions into the medical school curriculum, which revealed three themes: (1) desire to learn about burnout (students hoped to gain insight into burnout and methods of prevention as part of their curriculum); (2) importance of community (group interventions and the involvement of Faculty helped students feel less isolated in their experiences); (3) feasibility of interventions (sustainable interventions are likely to be those that are efficient, such as using multiple-choice questions, and with allocated periods in their timetable).

**Conclusion:**

Second-year medical students demonstrated symptoms and signs of burnout, including exhaustion, lack of motivation and changes in personality. They also expressed a desire to gain greater awareness of burnout and insight into preventative strategies within the medical curriculum. Whilst certain drivers of burnout can be prevented by students themselves through adequate prevention strategies, many remain systemic issues which require curriculum-level change to be effectively addressed. The students found that the reflection-based intervention was effective at improving their perception of burnout and a convenient tool to use, which could be implemented more widely and continued longer-term throughout medical school.

**Supplementary Information:**

The online version contains supplementary material available at 10.1186/s12909-023-04948-0.

## Introduction

Burnout syndrome is common amongst medical students [1, 2]. Previous work highlights that high levels of burnout are seen amongst medical students in pre-clinical and clinical years, resulting in feelings of professional inadequacy, poor academic engagement, higher intention to drop-out of medicine and suicidal ideation [1, 2]. With pressure in the United Kingdom’s National Health Service (NHS) at an all-time high [3] and increasing numbers of healthcare staff leaving the service [4], it is imperative to support the wellbeing and retention of our medical students. Similar phenomena are observed in health services internationally, especially following the COVID-19 pandemic, making this a globally pertinent topic [5].

Burnout has been described in the 11th Revision of the International Classification of Diseases as a syndrome resulting from chronic work-related stress that has not been successfully managed [6]. Burnout includes three components: feelings of exhaustion, negative or cynical feelings towards one’s work, and reduced efficiency at work [6]. Without adequate management, symptoms of burnout persist and deteriorate. Hence, designing methods to prevent and overcome burnout in medicine is essential for the health and safety of medical students and patients. A systematic review has demonstrated the need to implement interventions across all years of medical school [7]. Medical schools have started to implement such interventions with varying degrees of success, including education on the impact of work-related stress [8], encouragement of self-care practices, including exercise and spending time with friends or family [9], and mindfulness [7].

Reflection is an important practice in medicine. It has been introduced for stress and emotional management through practices such as Schwartz Rounds [10] and clinical reflection training [11] for health professionals and reflective writing for medical students [12]. Additionally, changes to the curriculum have been made to prevent burnout such as pass/fail grading [13], progress testing [14] and resilience curricula [15]. However, the impact of implementing burnout interventions and education into the medical curriculum is less clear.

A thorough understanding of the efficacy of interventions is needed to guide medical schools in helping their students prevent and manage stress. Therefore, the aims of this study are to gain insight into early year medical students’ experiences of burnout and appraise the value of a reflection-based intervention to support them in managing this. The objectives of this study are three-fold:

1. To understand the experiences of burnout and its underlying factors in second-year medical students.
2. To explore the impact of a reflection-based intervention on medical students’ awareness and experience of burnout.
3. To investigate the impact of implementing burnout interventions and education into the medical school curriculum.

## Methodology

### Study design

This study adopted an explanatory sequential mixed-methods approach. The intervention involved an anonymous online self-reflection task, including a questionnaire which generated quantitative measures of burnout in the cohort. The students were given three opportunities to fill out the self-reflection task. The intervention was introduced to the cohort in conjunction with two burnout teaching sessions that were implemented into the curriculum. This intervention was then followed by a focus group to explore the experiences and views of medical students in their second year of medical school at Imperial College London regarding burnout. A timeline of the methodology can be found in Additional file 1: Appendix 1.

### Implementations to the curriculum

Two sessions including content on burnout were designed and incorporated into the year 2 curriculum module *“Professional Values and Behaviours (PVB)”* (Additional file 1: Appendix 2). The year group consists of 345 people and in these sessions, the medical students were split into class sizes of ten to twelve:

- Session 1 (Oct 2020): The aim of this session was to improve students’ awareness of the presentation and drivers of burnout. Students were given two case-studies of students with burnout and were asked to reflect particularly on the signs and causes of burnout. Another aim was to destigmatise burnout by enabling students to find commonalities with the scenarios.
- Session 2 (Nov-Dec 2020): The aim of this session was to introduce a reflection-based intervention to students. They were shown a video, which discussed the self-reflection task, timeline, how the project will be conducted and a discussion of risks and benefits. This was supplemented by an information sheet and educational content about burnout.

The session materials can be found in Additional file 1: Appendix 3.

### Recruitment and participation

Participants were second year medical (MBBS) students at Imperial College London. All participants were over 18 years of age.

Students were invited to participate in the self-reflection task three times during the academic year, from November 2020 until March 2021. Voluntary response sampling was used to recruit students as they chose to participate by responding to the link in their PVB learning material.

1. Round one of the reflection-based intervention was completed between November to December 2020. This round was done after completing “Session 1” and in conjunction with “Session 2” described above. In this session students were specifically introduced to the research project through a video and information sheet.
2. Round two was completed between January to February 2021. There was no session to accompany this round, instead students were invited to participate by email or via an optional calendar invite.
3. Round three was completed in March 2021. There was no session to accompany this round, instead students were invited to participate by email or calendar.

At the end of the final PVB self-reflection task students were invited to opt-in to participate in a semi-structured focus group. It was necessary for the students to have participated in at least one round of the self-reflection task. The focus group was held in June 2021, after the summative examinations. Participation in all rounds of the self-reflection task and the focus group was optional, as was consent for their responses to be used in this research and did not impact their academic progression in any way.

#### Self-reflection task

The self-reflection task involved the completion of an online anonymous Qualtrics form, which was composed of three sections. The complete self-reflection task can be found in Additional file 1: Appendix 4.

#### Part one: revising the goals of medical students

This consisted of three free-text questions:

- What was my vision of the goal medical student that I want to become this year?
- What have I done this month to contribute toward this goal?
- What can I do next month to help me achieve my goal of medical student I want to be?

#### Part two: Shirom-Melamed Burnout measure (SMBM)

The measure is made up of 14 questions, split into three categories: physical fatigue, cognitive weariness, and emotional exhaustion. The wording was adapted for one question in the measure to enhance applicability for medical students, please see Additional file 1: Appendix 4 for the adaptation made. The use of this tool enabled a standardised way to measure and compare burnout scores between students, and to track student progression. It is also a relatively short tool, which may enhance student engagement, and has been shown to be effective in young people in gathering screening information with adequate validity [16]. Furthermore, this tool is free, which ensures that this is a low-cost intervention. Those participating received their own scores.

#### Part three: major stressor reflection

This section consisted of a single free-text question asking students to reflect on what the potential causes of their results from Part 2 might be:

- What are the main stressors that you have encountered over the past month?

#### Focus group

The focus group took place at the end of the academic year, following participation in all rounds of the reflection-based intervention. It was held online  using Microsoft Teams. This semi-structured discussion was designed to enable deeper exploration of participants’ views of their experience of burnout in medical school, their perception of the self-reflection task and suggestions of helpful implementations to the curriculum regarding prevention and management of burnout.

The focus group was facilitated by Imperial College teaching fellows, who were not associated with the study. The teaching fellows were inducted through a meeting and were provided with facilitator notes to support the moderation of the session.

Focus groups were audio and video recorded on Microsoft Teams; participants were invited to have their cameras off if they preferred to not be video recorded and to refer to each other using numbers to preserve anonymity. The focus group recording was transcribed verbatim by a third party (www.waywithwords.net) and participants were anonymised. The recording of the session was deleted upon transcription.

### Data analysis

#### Self-reflection task

Parts two and three of the reflection-based intervention were extracted from Qualtrics to an Excel spreadsheet. Responses for the SMBM (Part two) were converted into numbers on a scale from 1 (never) to 7 (always). Median and interquartile ranges were used to summarise the scores per round, per subtype of burnout (physical exhaustion, cognitive exhaustion, emotional fatigue) and overall. Although respondents responded to the survey more than once, only their first data response was accessible due to an error in the data collection system.

Free-text responses detailing key stressors amongst students (Part three) were summarised into distinct categories, which emerged from the responses. This analysis was performed by two researchers collaboratively and subsequently reviewed and discussed with a third researcher before the final themes were generated.

The frequency of each stressor category was calculated for responses overall and per round of the SMBM.

### Focus group

The focus group transcript was analysed on Nvivo 12.0 and the 6-stage methodology outlined by Braun and Clarke [17] was used as guidance for thematic analysis. A phenomenological inductive approach was used as it draws conclusions from participants’ own experiences, and thus aligns with the study’s aim to understand student’s experiences of burnout. Each transcript was coded by two authors independently (AMCP and MP) and discussed upon completion. A third reviewer (EM) was invited to revise the coding and resolve any discrepancies. Codes were devised into categories and later into themes following similar methodology.

### Consent and data protection

All students were provided with an information session and video that explained the risks and advantages of participation in the self-reflection task and focus group. Information sheets were also provided at the start of the reflection-based intervention and prior to the focus group. All information sheets highlighted that participation in this research was entirely voluntary.

All participants were asked for consent for their self-reflection task responses to be used in this research; they were also given the option to complete the reflection-based intervention without providing consent for research. Written, signed consent forms were required for participation in the focus group.

All data was stored on secure Imperial College London servers and anonymised.

### Ethics

The study was approved by the Imperial College Education Ethics Research Process (EERP) committee [EERP1920-103].

## Results

### Quantitative results

A total of 59 respondents to the SMBM consented for their data to be used, from a total of 101 students. 26 students participated in the first round of the measure, 8 in the second round and 25 in the third round.

**Fig. 1:**
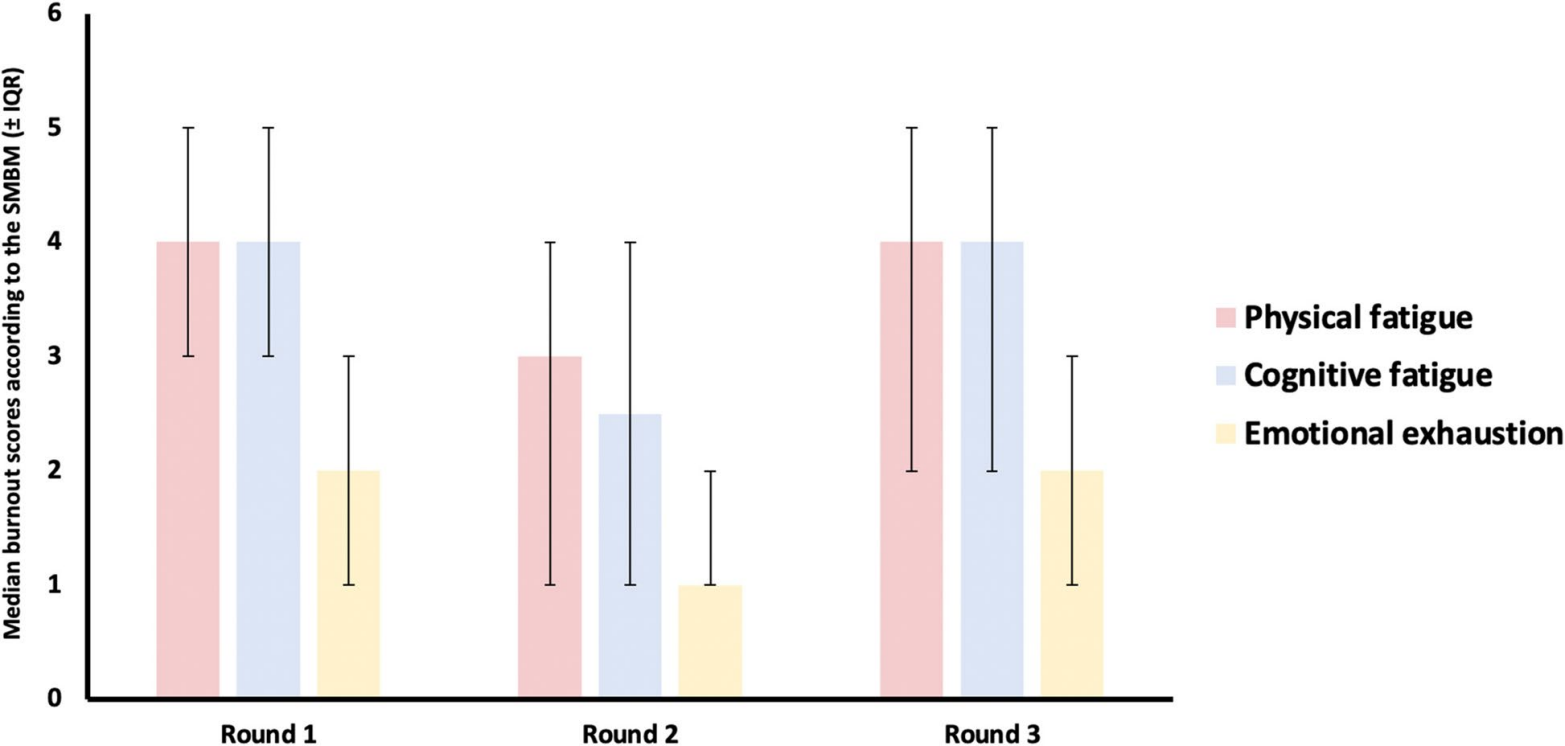
– median scores (± IQR) for physical fatigue, cognitive fatigue, and emotional exhaustion in each round of the Shirom-Melamed Burnout Measure (SMBM)

Overall median burnout scores were 4 (IQR 3–5), 2 (IQR 1–4) and 3 (IQR 2–5) in each round of the measure, respectively. Physical and cognitive fatigue consistently showed higher median scores than emotional exhaustion (Fig. 1).

Severe burnout has been defined as a mean score of 4.4 or above in the SMBM [18]. A total of 8 (30.8%) met this threshold in round 1 of the questionnaire, zero in the second round and 4 (16%) in the third round.

Stressors identified by students were categorised into 5 groups: academic, mental health, physical health, social life, Coronavirus Disease 2019 (COVID-19) pandemic, with the remainder labelled ‘other’ (see Additional file 1: Appendix 5 for definitions and full list of stressors under each group).

**Fig. 2:**
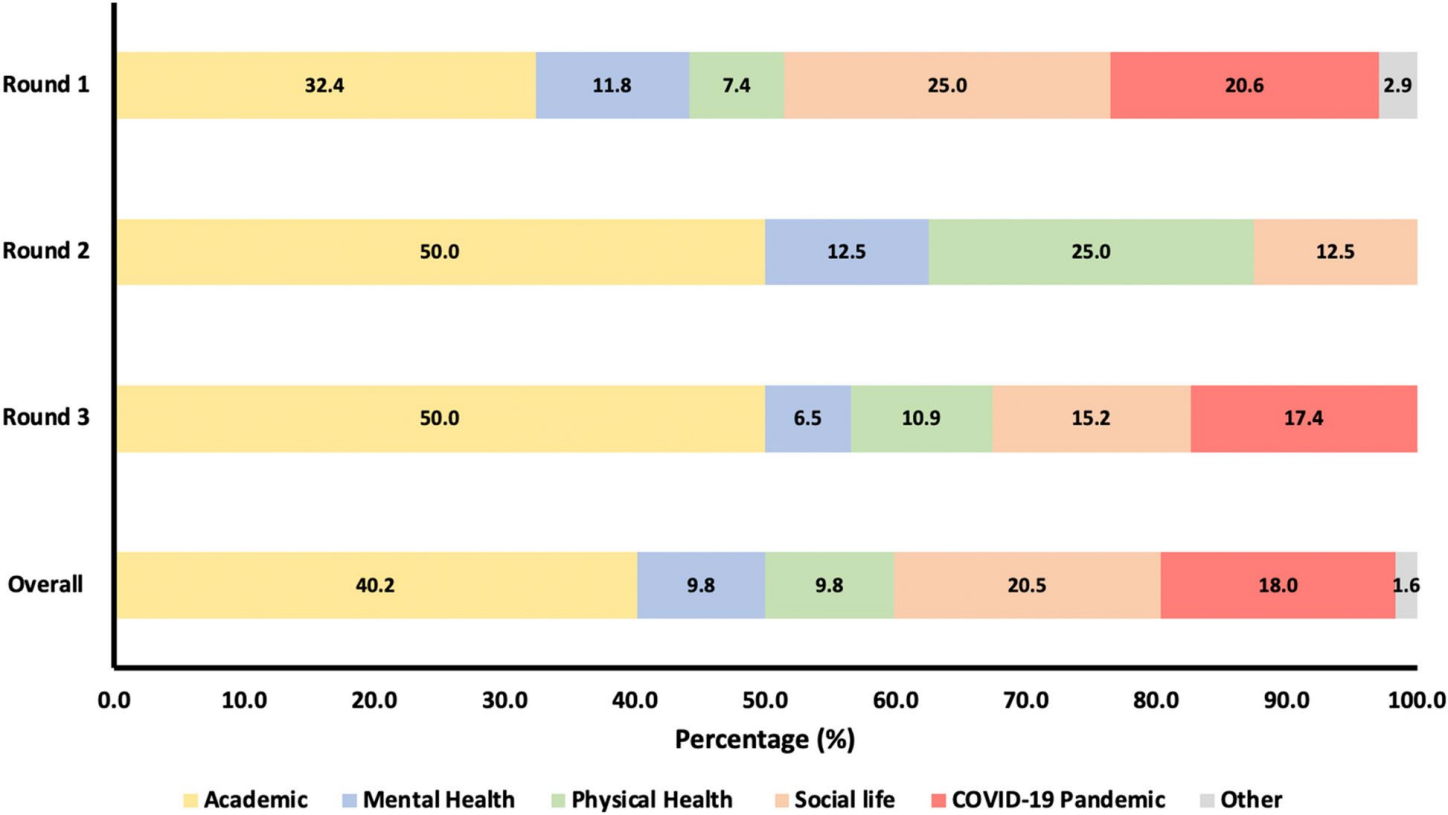
– Groups of stressors as a percentage of total identified stressors for each round of the reflection-based intervention and overall (n_1_ = 68, n_2_ = 8, n_3_ = 46, n_overall_ = 122)

Academic-related stressors were those most frequently mentioned in all rounds of the intervention (Fig. 2). Social life-related stressors were the second most mentioned overall, followed by the COVID-19 pandemic.

### Qualitative results

Four students participated in the focus group. Focus group findings were grouped into two sections, each with their independent themes.

### Reflecting on burnout in medical school and the intervention

This section captures what drives burnout in medical school and how it is experienced by medical students. Furthermore, it evaluates the impact that the intervention had on this group of medical students. Four themes were identified in this section.

### Indicators of burnout in medical school

There was consensus that students are often unaware of their burnout and don’t take the time for self-reflection. Students commented that it would often be others who would recognise their signs of burnout.

“*A lot of the time, because you’re busy and you’re stressed you don’t think about the fact that you’re busy and you’re stressed. Which then makes everything worse, it’s like a vicious cycle.” (B1)*

The term “*insidious*” *(A1)* was used to describe the manifestations of burnout: slowly impacting students’ lives but not noticeable until one cannot continue with life as normal. As one student explained:

*“I’m starting to approach my limit now because I’ve been working solidly for the last few months, and it is without a break… weekends aren’t really weekends…and evenings aren’t really evenings because you’re working. It’s that build-up and you feel it towards the end of term.” (B2)*

One of the signs of burnout noticed is a disconnect from themselves, described as *“personality change” (A2, B3).* Students described this as a feeling of being able to go about your day-to-day but having a different attitude towards it.

*“I’m still doing the work, and I’m still going through the motions, but… I just don’t really feel like me.” (B4)*

This “personality change” was accompanied by a change in emotions and behaviours, including cynicism and frustration towards themselves:

*“Yes, that feeling of frustration is definitely a big thing, that feeling of being inadequate because you think oh, I was able to do this before, why can’t I do it now? What is wrong with me.” (A3)*

Students reported having less motivation and energy when burnt-out; an “*underlying feeling*” *(B5)*, creating a sensation of “*brain fog*” *(C1)*. Specifically, they notice losing their enjoyment for medicine.

The focus group prompted students to reflect on coping strategies they use to manage burnout; exercise was most frequently mentioned followed by going outdoors and music. Hobbies were described as reminders of “*life outside medicine*” *(D1)* that bring a “*sense of focus and calm*” *(C2)*. However, participants also described that often their way to cope was to “*crack down and do the work*” *(B6)*.

### Perceived drivers of burnout at medical school

Some drivers of burnout exist even before students begin medical school; students enter medical school with a sense that this will be “*one of the most stressful experiences to go through” (A4)*. Students explained that this is reinforced during medical school interviews, where students aim to “*prove that you are invincible*” *(C3)*, emphasised by questions such as “*how well can you cope under pressure?” (C4)*.

*“It’s almost an expectation entering into this career that you should be able to deal with the abnormal, and you shouldn’t have signed up to do this if you weren’t under the expectations of what would be ahead. That it’s normal that you should be able to just deal with it” (C5)*

A recurring theme in the student narratives was that being a doctor means being *invincible (C3, B7).* Students perceived constant and overwhelming work to be a characteristic of a doctor’s job. There was a recurring idea that doctors should be able to “*put their stress to one side” (A5)* and “*just carry on” (A6).* This is demonstrated in the quote below:

*“But it’s that underlying thing of you have to be invincible if you want to be a good doctor because you have to be able to work 100-hour weeks and never feel any element of stress or burnout” (B8)*

Students explored that this is subconsciously influenced by observing healthcare workers on placements and systemic issues within medical school. For instance, students described that stigma is exacerbated by hearing doctors’ stories of their “*thankless job” (A7)*. Within medical school, it was acknowledged that the fast pace, encouraged by their timetable, emphasises an idea that everyone should be able to *“handle constant work” (B9).*

*“So, you assume that it’s normal to have a scheduled day nine till five and then have a load of work to do before and a load of work to do after…you should be able to handle it. And you don’t clock that actually maybe everyone struggles to handle that sometimes when it gets a bit too much” (B10)*

Students also described struggling to dedicate time to reflection. They did not feel that they had to recognise burnout, despite an awareness that it would help their wellbeing. Students said or commented that burnout is a *“taboo” (D2, B11)* topic, encouraged by observations of doctors who “*get on with it*” *(A8)* despite signs of burnout.

Students also felt a sense of isolation in their experiences of burnout at medical school. This was partly due to perceiving their peers to be succeeding, whilst they are struggling with burnout. Additionally, there was a pattern that during particularly stressful times, students would resort to limiting their social contact. The reasoning tended to be that they wouldn’t “*allow*” *(C6)* themselves social contact if they could still be working. Thus, isolation seems to be not only in association with burnout but also a resulting behaviour of it.

### Working habits of medical students

Students displayed very high expectations of themselves. The underlying driver behind these expectations was often perfectionism, with frequent references to “*wanting to do the absolute best” (A9).* Simultaneously however, students appreciated that these expectations were often unrealistic, creating conflict between their reality and their self-expectations:

*“You’re never going to walk into every single exam and think I’m 100% sure that I’m going to ace this and I’m going to get full marks because I know absolutely everything. And I’m a bit of a perfectionist and so, I’ll think I want to get as close to perfect as I physically can” (B12)*

As a result of these expectations, students displayed harshness towards themselves. For instance, a student described how “*I don’t deserve to relax because there’s definitely a list of things I could still be doing right now” (A10).* There is guilt associated with not working, often meaning students struggle to engage in activities that they enjoy outside their studies.

“*I won’t stop until after the exam. Because I always think I could be that bit better. And I always think if I do half an hour of Anki right now rather than watching half an hour of Netflix before bed, I might get an extra 1 or 2% and that could make a difference. And so, that’s always in the back of my mind. So, I can’t enjoy switching off, so I’m like, what’s the point of switching off?” (B13)*

Students widely acknowledged that there is always more to learn in medicine, as “*there being no end to it*”. Despite awareness that they can never learn everything, students do not know when to stop, resulting in an unwillingness, or inability, to take breaks.

*“There’s no end to your revision, so you just don’t stop. So, it’s not a case of you have time during the day where you switch off, it is just constant” (B14)*

Students also highlighted the culture in medical school as productivity-orientated - if they do not believe that something is contributing to their work, then they are often unwilling to engage. This makes it difficult for many prioritise their wellbeing. Students recognised that an “*attitude shift” (A11)* was required to emphasise the value of practices such as self-reflection.

#### Value of the intervention

Students acknowledged the intervention as valuable – particularly, they described the self-reflection task as the most helpful tool in prompting them to reflect and identify symptoms of burnout within themselves. Prior to this, many did not realise they were burnt-out. This also resulted in a newfound appreciation for reflection.

*“I’ve learned more about myself and identified more aspects of stress and burnout within my own life than I thought I would have. I didn’t think about it before, which is what we were saying a lot, is you don’t stop and think and identify it… I certainly didn’t expect to actually identify as many things and relate to it as much as I did.” (B15)*

The intervention prompted students to consider strategies to prevent burnout by identifying habits which contribute towards burnout. This helped them to either adapt or build new habits, such as self-reflection which was often spoken about as a new coping strategy.

*“…just stopping to think about it is useful, because you do suddenly take the time and identify it. And in the long run, it’s a lot more helpful to stop and to think about it and identify it and think about ways to tackle it… So yes, it’s definitely worth doing.” (B16)*

### Considerations for implementing burnout interventions into medical school curriculum

The second section focuses on considerations for implementing burnout interventions into the medical school curriculum. Three themes are described which explore the facilitators and barriers of implementing burnout into the medical school curriculum and more specifically, the intervention. These also capture specific considerations for any intervention highlighted by the students.

#### Desire to learn about burnout in medical school

Students noted that the place they have learned most about stress and burnout is within the classroom. They valued the opportunity to learn about burnout in this context and suggested they would like this *“incorporated across modules” (A12).* Specifically, students mentioned a desire to learn more about the pathophysiology behind burnout and the reasoning behind coping mechanisms.

*“It gives me more of a reason to think about when I’m sleeping, when I’m eating, the circadian rhythm, getting sunlight. How the knowledge of the science behind that was more what helped me provide a reason to make those lifestyle changes” (C7)*

Furthermore, students hoped that teaching would support them in developing personalised strategies to implement these changes into their lives. They also highlighted the importance of being encouraged to enact this change. One strategy suggested was being given allocated time to engage with burnout-prevention strategies such as *“having protected time to be able to go outdoors” (A13).*

Finally, students also mentioned a desire to understand how the university uses information shared in any surveys, reflections or other forms of feedback during teaching. *“It would be more motivating if we were made aware of how our reflections and how our results are being used by the Faculty to make changes” (C8).*

#### Importance of community

A recurring theme within students’ narratives was the desire to be in a community. This was conceptualised as an understanding that you are not alone in struggling with burnout. Therefore, students appreciated acts such as students participating in the intervention together in a lecture theatre.

*“And the biggest consolation for me personally…when other people are like, don’t worry, I feel the same way. That sense of not being alone in that situation, that’s probably one of the big things, getting students to understand…it is common for that to happen” (A14)*

Student accounts revealed a conflict between a desire to seek help from their community but a fear of appearing as a failure. Their narratives revealed that students believed that asking for help about burnout would mean that they “*would not be successful in their medical career”* or that they “*shouldn’t be in medical schoo*l” *(C9, B17)*. They reported that destigmatising burnout in medical school would encourage them to seek help.

Faculty members were widely recognised as figures of trust in the medical school. Student accounts showed that if Faculty members would speak about burnout, then they would find greater reassurance that it is common. If staff encouraged coping strategies, then students would be more likely to try them. This is portrayed in the following quote:

*“The fact that my faculty members are telling me to do these things has helped me realise the importance of it” (D3)*

#### Feasibility of interventions

Whilst students noted the importance of encouraging healthy lifestyles to prevent burnout, they highlighted the importance of making these *“feasible to manage with [their] day-to-day life” (D4).*

Students suggested that proposed changes or interventions should be implemented gradually, *“starting with baby steps and incorporating a small, manageable chunk (…) and as you get more used to having that as part of your routine, then you’ll be more willing to increase the amount of time that you dedicate to it” (A15).* Otherwise, students may be less willing to engage with interventions.

## Discussion

Overall, quantitative results suggest a moderate level of burnout within the cohort (median score 4 out of 7), with relatively higher levels of physical and cognitive fatigue. Highest levels of burnout were seen in the first round of the intervention, with 30.8% of participants showing severe levels of burnout (≥ 4.4). Focus group data suggests that the reflection-based intervention and teaching sessions helped medical students gain insight into their experiences of burnout and consider helpful management strategies.

The burnout literature confirms that academic factors are a major source of stress and driver of burnout amongst medical students, including high workload and challenges with time management [19, 20]. Students in this study however, showed awareness of these stressors and of strategies to help manage and prevent burnout, including investing time in social relationships, engaging in sports and hobbies, and taking breaks from work. However, this study was performed during the COVID-19 pandemic, which has itself challenged students’ ability to practice many of their hobbies and changed their social relationships and studying habits [21]. Student mental health and anxiety also worsened during the pandemic [22, 23]. This therefore raises concern over the long-term impact of the COVID-19 pandemic on burnout amongst medical students. Burnout has been found to be associated with increased risk of suicidal ideation, further emphasising the importance of supporting students in preventing and managing their experience of burnout [24]. Whilst a systematic review of studies from medical schools in China also confirmed the importance of social support as a protective factor against burnout itself, they also highlighted the value of institutional support [25]. Therefore, as the world grows out of the pandemic, the need for clearly signposted, accessible sources of support within medical schools remains vital.

Further literature notes the importance of mindsets and cognitive processes on the development of burnout. Resilience has repeatedly been shown to be an important characteristic in medical professionals to support them in managing emotion at work and prevent burnout, including during the COVID-19 pandemic [26–28]. Similarly, adaptive coping strategies and ‘grit’ have both been associated with lower levels of burnout [29, 30]. These are coping mechanisms that may be developed through practice and reflection – which were not initially discussed as strategies to manage burnout by medical students in this study, suggesting lack of awareness of  their value and importance. It may therefore be valuable for medical schools to support students in developing personal strategies to prevent and manage burnout, including methods of reframing and ‘growth mindsets’ [31]. However, it is equally important to identify preventable drivers of burnout outside the students’ control and support students through efforts to resolve these issues. Results also highlighted the continued perception of the doctor as a hero: one that must take on everything without bearing the burden. Consequently, students revealed feeling pressure to continue performing as exemplary students through all challenges, including illness and exhaustion, a concept that may be perpetuated by the emphasis of resilience within medical curricula. A clear distinction must be made between resilience and the detrimental concept of “invincibility”.

Integrating burnout teaching into the curriculum equips students with the fundamental knowledge to identify, prevent and manage burnout early on. Combining this with a screening tool enabled greater self-awareness of personal experiences of burnout in an accessible, cost-free way [32, 33]. The students in the study highlighted that the screening tool provided a quick and practical introduction to reflection. Furthermore, it aligned with a model of behaviour change where small incremental changes, such as screening tools, can build to form long-term habits, such as reflection [34, 35].

One of the barriers to engaging with burnout prevention and management is prioritization of academic study [19, 20]. Students in this study revealed that despite being aware of strategies to help manage and prevent burnout they experienced various self-imposed and societal expectations with regards to academic achievement that stopped them from prioritising their wellbeing, and instead forcing them to focus on their studies. The integration of learning about burnout within academic study may help to overcome this barrier. Results also identified that students with burnout symptoms are reluctant to ask for help due to a fear of failure; implementing burnout into the curriculum also provides students an easy way to access educational material and opportunities to encourage asking for help.

Additionally, participants in this study highlighted the value of learning about and reflecting on their burnout experiences in groups. This enabled students to recognise the experience of similar feelings amongst their peers, providing a sense of community and support by reassuring them that they are not alone in their experience of burnout and additionally made them feel more comfortable to ask for help. The use of peer-support groups and group problem-based learning has been trialled previously for burnout prevention, with participants reporting several benefits including greater sense of belonging, self-confidence and general appreciation for speaking with others in the same situation [36]. Shared reflection has also been used successfully in Schwartz Rounds at Imperial College, which showed an improved sense of support and were found to be more valuable than written reflection [10]. Therefore, it is important to consider building a community in the teaching of burnout.

Group learning also encouraged the de-stigmatisation of burnout, with results suggesting that this would be particularly effective through the involvement of staff in burnout teaching. Students showed an inherent trust in faculty members and stated they were more likely to engage in burnout prevention if encouraged by staff. Literature has previously suggested the value of supervisor support [24] and the potential of mentorship programmes [37] in preventing and managing student burnout by increasing students’ sense of emotional and professional support [38]. This may be likened to the existing academic tutoring programme at Imperial College, which was also highlighted in focus groups as an important source of support for students experiencing stress and burnout.

The session highlighted the importance of burnout awareness to students and established the idea that this can be incorporated into a medical curriculum. It introduced material important to both students and the patients they will treat. In future, the intervention could incorporate more effective ways of engaging students in responding to the self-reflection task, such as filling it out in the presence of the facilitator of a teaching session. Furthermore, an element of group reflection could be incorporated into the self-reflection task, such as a group discussion to destigmatise the stressors of medical school. Finally, the impact of completing the self-reflection task in regular intervals should be measured.

### Limitations

There are limitations of this study that should be acknowledged. Despite findings aligning with broader literature, the size of the sample is insufficient to ensure it is representative of all second-year students. Responses are also not paired, meaning that it is difficult to be certain that changes seen in the intervention results over time are illustrative of the reality for this cohort of medical students. Furthermore, a sensitivity analysis could have been conducted to sensitise the sample to burnout and reduce the impact of confounding factors. Whilst the focus group enabled views and experiences to be explored in greater depth, the sample of participants is likely biased – that is, those more likely to be interested in learning about burnout and self-development, or perhaps more affected by burnout. Similarly, it is possible that the intervention itself may have sensitised students to burnout – whilst helping students become more aware of signs of burnout in themselves is arguably a positive impact of the intervention – it might mean that students who did not participate in the intervention may not yet entirely relate with these experiences. It is also important to highlight that this study was performed over 4 months; burnout often has an insidious onset, and identifying and managing stressors may take a much longer time. In the future, studies should consider a longer follow-up period. The COVID-19 pandemic also represents an exceptional time for everyone, including medical students, and hence may have impacted perception and experience of burnout. All data is also self-reported, which relies on the assumption that students were honest when answering questions. Despite the reassurance that responses were anonymized, some may still not have felt comfortable to report particularly negative experiences.

## Conclusion

Medical students begin showing signs of burnout as early as the first years of medical school. There is a desire to learn about burnout and effective coping mechanisms, however academic studies often take precedence. The study verified that burnout prevention and management should be part of the academic curriculum, whilst highlighting the value of frequent, short self-reflection tools as a means of achieving this in an accessible way and the importance of establishing supportive communities in medical school. Finally, this study opens discussions around the idea of doctors as “invincible” “heroes” and possible impacts of this on medical students, which need to be explored further. Future studies should also investigate the use of simple reflection-based exercises in large sample sizes and the effect over time.

### Supplementary Information

**Additional file 1.** 

## Data Availability

The datasets used and analysed for the study are available from the corresponding author upon reasonable request.
