# Supplementary material for: Burnout in early year medical students: experiences, drivers and the perceived value of a reflection-based intervention
Source: BMC Med Educ. 2024 Jan 3;24:7. doi: 10.1186/s12909-023-04948-0 (PMC10765935; doi:10.1186/s12909-023-04948-0)
Supplement: Supplementary file 1 — Additional file 1. [file 12909_2023_4948_MOESM1_ESM.docx]

**Appendix**

**Appendix 1**

Timeline of the Methodology

**
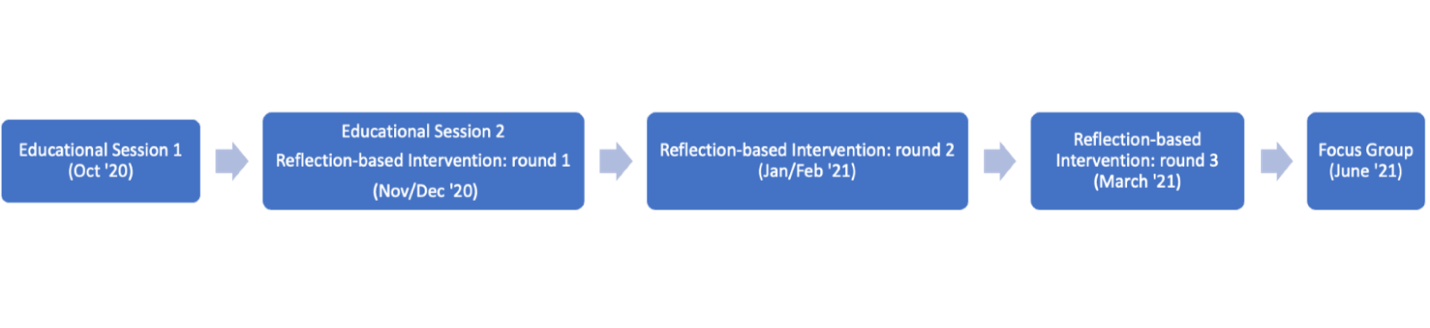
**

**Appendix 2**

Description of the Professional, Values and Behaviours Domain

Teaching and learning sessions about being a medical student and becoming a doctor are delivered in a spiral curriculum across the six years of our course. More details are available on request ([e.muir@imperial.ac.uk](mailto:e.muir@imperial.ac.uk)).

**Appendix 3**

Session Materials for the Methodology

**Participant Consent Form**

Investigating and preventing burnout in medical students through the use of a self-reflection task

Investigator: Ms. Mabel Louise Prendergast

Principal Investigator: Dr Elizabeth Muir

Start Date: 27^th^ November, 2020 End date: 30^th^ April, 2021

Please create a unique ID number using the last 3 letters of your surname and the last 3 digits of your phone number

__ __ __ __ __ __

Please enter your email. This will only be used if we flag an extremely concerning response in your answers and feel you need to be contacted and put in contact with our welfare team.

_________________________________________________

YES NO

I have read and understood the participant information video and sheet

about the project

I am satisfied with the explanation of the risks and benefits of the project

The risks and benefits have been discussed

I agree to provide my anonymous information as outlined on the information

sheet

I understand that my participation is voluntary and that I can withdraw from

the project without giving any reason, and that if I choose to do this, any data

I have contributed will not be used

I understand that I can contact Ms. Mabel Prendergast at any time

I consent to take part in the study

**Information Sheet** (*details have been excluded for privacy reasons and are replaced with “x”)

Rationale

Welcome to your self-reflection task. There are three parts to this self-reflection task. Firstly, you will revise your vision from the Professional, Values and Behaviour session, of the goal medical student that you want to become. Then you will be asked to self-reflect on experiences of stress that you may have encountered over the past month. Lastly, we would like you to think about the stressors in your life. You are being asked to pay close attention to your inner thoughts and interpret this as a practice of mindfulness. These exercises will be supported by curriculum content from the PVB and LMAP sessions.

What do I have to do?

You will be completing this task online every 4 weeks over a period of 4 months from the end of November to April. Please do remember that we strongly recommend that you complete this by yourself and keep your answers private. We also recommend that you complete this task consistently. In order to be reminded of when to complete this task, subscribe to the iCalendar using the following links:

HTML: x and ICS: x

What are the risks and benefits?

We hope for this self-reflection task to enable you to recognise the symptoms of stress in yourself and learn how to manage them in a *positive* manner. You will find this exercise regardless of whether you feel that you are stressed or burnt out. The purpose is to benefit the mental health of the student body as we are seeking to understand the potential causes of burnout.

This task will not only be personally useful but will also help you in future patient encounters. Burnout is a problem that is facing a lot of the working population and therefore also many of the patients you will meet.

We understand that an analysis of burnout symptoms and stressor identification can be an uncomfortable task for some of you. When paying attention to thoughts that you might otherwise ignore, you may find yourself in a difficult position. However, we have many support networks that are always available for you if you may find yourself in such a position.

- Your personal academic tutor
- Your senior tutor: x
- FEO welfare: x
- Student Union Welfare:
- Your welfare representative

How will my data be handled?

Your data is anonymous and is being collected in a GDPR-friendly manner. We are asking you to provide an email that we can associate with your ID for safeguarding. This information is being kept separately from your data and will only be accessed in the case of a concerning response that we flag in our data analysis.

How do I find out more?

You will always be able to contact me for any questions, concerns, or ideas you may have. My email address is x.

I look forward to working with all of you in the next few months and I sincerely thank you for your participation in this project. I am very eager to see where this can take us!

Best wishes,

Mabel Prendergast

**Appendix 4**

*The adaptation has been highlighted

**Self-Reflection Task**

If you have any welfare concerns then please contact your personal academic tutor, your senior tutor, FEO welfare, your welfare representative or the SU welfare team.

**Medical Student Goal**

Part 1 of the task is referring to the medical student that you envisioned yourself to become in the Professional Values and Behaviours Session “professional working:professional boundaries”. If you feel that you are not making progress to fulfilling your goal, consider revising it to suit your lifestyle and wellbeing.

What was my vision of the goal medical student that I want to become this year?

What have I done this month to contribute toward this goal?

What can I do next month to help me achieve my goal of medical student I want to be?

**Shirom-Melamed Burnout Measure**

Part 2 of the task is asking you to self-reflect on your symptoms of stress and potentially burnout. Please remember that there is no right or wrong response when answering these questions, it is simply an honest reflection of you. It is important to follow your first instinct. All answers are being kept anonymous.

I feel tired

I have no energy left to attend university in the morning

I feel physically drained

I feel fed up

I feel like “my batteries are dead”

I feel burnt out

My thinking processes have slowed down

I have difficulties concentrating

I am not thinking clearly

I feel that my thinking is not focused

I find it difficult to think about complex things

I feel that I am unable to be sensitive to the needs of others

I feel that I am unable to emotionally invest myself in other people

I feel that I cannot sympathise with other people

**Stressor Identification**

Part 3 of the task is an open question asking you to reflect on what the potential cause of any of the symptoms from Part 2 may be.

What are the main stressors that you have encountered over the past month?

**Appendix 5**

**Definitions of stressors**

**Academic**

This stressor has two components. Firstly, it encompasses the workload faced in medical school including exams and virtual learning. Secondly, it encompasses the expectations that student’s put on themselves because of the workload.

**Mental Health**

This stressor incorporates the mental health concerns of medical students. This includes both mental health conditions such as eating disorders and anxiety as well as stressors that can negatively impact mental health such as loneliness and body image.

**Physical Health**

This stressor encompasses most components of a medical student’s physical health including the categories of sleep, nutrition, physical activity, and physical health conditions.

**Social life**

This stressor concerns the social interactions of medical students with their family, friends, and partners. Included within this category is their extra-curricular activities and how they can balance this with medical school.

**COVID-19 Pandemic**

This stressor includes all stressors related to COVID-19. This includes academic concerns such as difficulty focusing and virtual learning. However, it also includes the social aspects of reduced social interaction, a different pace of life and the fear of COVID-19.
 **Other**

Included in this category are student finances and moving to new environments.
